# Optimising multiple myeloma therapy in resource-limited settings: current perspectives and challenges

**DOI:** 10.3332/ecancer.2025.2005

**Published:** 2025-10-06

**Authors:** Bishal Tiwari, Samita Sapkota

**Affiliations:** 1 Nassau University Medical Center, East Meadow, Long Island, NY 11554, USA; 2 Manipal Teaching Hospital, Pokhara, Kaski 33700, Nepal; a https://orcid.org/0000-0002-5145-1448

**Keywords:** multiple myeloma/therapy, multiple myeloma/diagnosis, health resources, health services accessibility, developing countries, healthcare disparities, cost-benefit analysis, health policy, global health

## Abstract

Multiple myeloma (MM) represents a significant global health challenge, with its incidence and mortality rates steadily increasing over recent decades. This review critically examines the current landscape of MM management, with a specific focus on resource-limited settings, where the disparities in diagnostic capabilities and treatment options are most pronounced. A comprehensive literature search was performed using multiple databases, encompassing peer-reviewed articles, clinical guidelines and conference abstracts from 2010 to 2024. Our analysis delineates the stark differences between therapeutic approaches in high-income versus low- and middle-income countries (LMICs). In high-income settings, the standard of care involves advanced induction regimens, autologous stem cell transplantation and maintenance therapy with novel agents, which collectively have contributed to improved patient outcomes. Conversely, LMICs often rely on more affordable yet less effective treatments, such as bortezomib- or thalidomide-based regimens, largely due to limited access to advanced diagnostics and high-cost therapies. Key challenges identified include late presentation, inadequate diagnostic infrastructure, economic constraints and a paucity of trained healthcare personnel. To address these issues, we propose a multifaceted strategy that emphasises the enhancement of diagnostic capacity, the adaptation of resource-stratified treatment guidelines and the strengthening of healthcare systems through targeted policy interventions and international collaborations. By bridging the gap between evidence-based MM care and the practical realities of under-resourced healthcare systems, this review aims to inform future clinical practice and policy, ultimately improving survival outcomes and reducing global health inequities in MM management.

## Introduction

Multiple myeloma (MM) is a plasma cell neoplasm characterised by clonal plasma cells that produce a monoclonal immunoglobulin. These plasma cells proliferate in the bone marrow and can result in extensive skeletal destruction with osteolytic lesions, osteopenia and/or pathologic fractures [1]. The global burden of MM has risen significantly, with increasing incidence, mortality and disparities in the quality of care across different regions of the world. The incidence of MM increased by 136.1% between 1990 and 2019, with an age-standardised rate of 1.92 per 100,000 population [2]. In high-income regions such as North America, the prevalence rose from 42,637 cases in 1990 to 101,994 in 2019, with deaths increasing from 11,200 to 19,941 during the same period [3]. Similarly, in China, MM cases reached an estimated 18,793 in 2019, with disability-adjusted life years rising significantly from 148,479 in 1990 to 347,453 [3].

Even though the global quality of care index for MM improved from 51.31 in 1990 to 64.28 in 2019, disparities persist, particularly in low-income countries (LICs) [4]. The prognosis of patients with MM has improved significantly in the past two decades. This is attributed to the use of novel agents for induction, high-dose chemotherapy and autologous stem cell transplantation (ASCT), maintenance therapy and improved supportive care. Currently, evidence-based management guidelines/recommendations developed by international societies/groups are being followed partially in low-resource settings. However, lack of quality diagnostics (e.g., cytogenetics/fluorescence *in situ* hybridisation, serum free light chains), novel therapeutics and trained manpower and limited financial resources are key constraints leading to disparities in care.

Management of MM in low- and middle-income countries (LMICs) represents a big challenge for healthcare providers considering the economic, technological and infrastructural restraints in comparison to developed countries. Context-specific strategies are required for successful evidence implementation in LMICs and a number of common barriers can be addressed using locally available, low-cost resources. Education for healthcare workers in LMICs is an effective awareness-raising, workplace culture and practice-transforming strategy for evidence implementation. The delivery of evidence-based treatment for MM in LMICs faces significant barriers, primarily due to resource limitations and systemic healthcare challenges [5, 6]. Addressing these issues requires a multi-faceted approach to improve access, delivery and education [7].

This analytical review explores contemporary evidence on barriers undermining myeloma treatment efforts in resource-constrained LMIC settings. We conducted a comprehensive literature review of peer-reviewed evidence on myeloma control challenges and solutions tailored to resource-limited settings. This review aims to explore evidence-based strategies to optimise the treatment and maintenance of MM in resource-limited settings. It seeks to address the unique challenges faced by LMICs, where disparities in access to therapies, diagnostic tools and supportive care are prevalent. The significance of this review lies in its potential to guide clinicians, policymakers and researchers toward actionable solutions that bridge the gap between evidence-based MM care and the limitations of under-resourced healthcare systems. In doing so, it aims to improve survival rates, enhance quality of life and reduce global health inequities in MM care.

## Methodology

### Literature search strategy

A systematic review was conducted to identify relevant literature on optimising MM therapy in resource-limited settings, with a focus on treatment and maintenance strategies. The search included peer-reviewed articles, guidelines and conference abstracts published in English between January 2010 and December 2024. A total of 92 studies were included in the final synthesis. These comprised of 48 peer-reviewed original articles (including retrospective studies, observational cohorts and real-world data reports),18 review articles or consensus guidelines, 12 conference abstracts from ASH/International Myeloma Working Group (IMWG)/European Society for Medical Oncology (ESMO) and 14 government and institutional reports relevant to health policy and drug accessibility in LMICs.

The following databases were queried: PubMed, EMBASE, Web of Science, Cochrane Library. The search strategy employed combinations of Medical Subject Headings terms and free-text keywords, including: ‘MM’, ‘resource-limited settings’, ‘LMICs’, ‘treatment strategies’, ‘maintenance therapy’ and ‘cost-effective alternatives’.

Although this is a narrative review, we adhered to key principles of transparent literature synthesis and structured our process in alignment with elements of the PRISMA 2020 checklist where applicable. This includes explicit inclusion/exclusion criteria, dual screening of abstracts/titles and data abstraction focused on treatment access, outcomes and resource constraints. References of included articles and key review papers were manually screened to identify additional relevant studies.

### Inclusion and exclusion criteria

#### Inclusion criteria

Studies addressing MM treatment or maintenance therapy in resource-limited settings.

Articles discussing cost-effective adaptations or challenges in accessing standard MM therapies.

Clinical guidelines and reviews highlighting innovative approaches to MM care in LMICs.

Observational studies, randomised controlled trials and meta-analyses.

#### Exclusion criteria

Studies exclusively focused on high-income countries (HICs) without consideration of resource limitations.

Non-english publications and articles with insufficient data on MM management strategies.

Case reports and editorials not providing actionable insights.

The extracted data were synthesised to highlight common themes, challenges and potential strategies for optimising MM care in resource-limited settings.

#### Risk of bias assessment

Given the heterogeneity of included sources (especially, real-world studies and country-level reports), formal risk-of-bias scoring tools (e.g., ROBINS-I or Cochrane tools) were not uniformly applicable. However, we categorised included studies based on their design quality and flagged limitations (e.g., single-center design, lack of control group and incomplete follow-up) in our synthesis.

## Results

### Comparison of current treatment strategies for low and HICs

#### Treatment options for MM in LICs

Available options are often constrained by limited resources, including access to novel therapies and advanced diagnostic tools. However, several strategies can be employed to manage MM effectively within these constraints.

First-line treatment typically involves the use of bortezomib-based regimens or thalidomide-based regimens due to their relative affordability and availability. Bortezomib, a proteasome inhibitor, is commonly used in combination with dexamethasone and cyclophosphamide bortezomib, cyclophosphamide, dexamethasone (VCD) or with thalidomide and dexamethasone (VTD). Thalidomide, an immunomodulatory drug, is often used in combination with dexamethasone and cyclophosphamide (CTD) due to its lower cost [8, 9]. We note that while thalidomide-based regimens (e.g., VTD or CTD) are still commonly used in many LMICs due to broader affordability, bortezomib, lenalidomide, dexamethasone (VRd) has become the preferred first-line regimen in India and some middle-income nations where cost-effective generic lenalidomide is accessible [10].

Maintenance therapy frequently involves thalidomide due to its affordability and effectiveness in prolonging remission [8]. ASCT, while a standard of care in HICs, is less frequently performed in low-income settings due to its high cost and the need for specialised facilities [9, 11].

For relapsed or refractory MM, bortezomib-based regimens remain a preferred option, although their use may be limited by cost. More affordable alternatives include cyclophosphamide, thalidomide and dexamethasone (CTD) [8]. Supportive care is crucial and includes the use of bisphosphonates for bone disease, antibiotics for infection prophylaxis and blood transfusions as needed [11]. Efforts to improve MM management in LICs should focus on increasing access to generic versions of effective drugs, enhancing diagnostic capabilities and improving healthcare infrastructure [12].

#### Treatment options for HICs

The standard treatment regimen for MM in HICs involves a combination of induction therapy, ASCT for eligible patients and maintenance therapy.

For transplant-eligible patients, the induction therapy typically consists of a triplet regimen such as VRd for 3–4 cycles. In high-risk patients, the addition of daratumumab to this regimen (Dara-VRd) is recommended. Following induction, patients undergo ASCT, which is then followed by maintenance therapy with lenalidomide for standard-risk patients or a combination of bortezomib and lenalidomide for high-risk patients [13–15].

For transplant-ineligible patients, the standard induction therapy includes VRd for approximately 8–12 cycles or daratumumab, lenalidomide and dexamethasone until disease progression. Maintenance therapy with lenalidomide is recommended for standard-risk patients, while high-risk patients may benefit from a combination of bortezomib and lenalidomide [13–15].

The American Society of Clinical Oncology (ASCO) and the ESMO guidelines support these treatment approaches, emphasising the use of triplet or quadruplet regimens for induction and the importance of maintenance therapy to prolong remission and improve survival outcomes [13, 16, 17].

In summary, the standard treatment regimen for MM in HICs includes induction therapy with VRd or Dara-VRd, ASCT for eligible patients and maintenance therapy with lenalidomide or a combination of bortezomib and lenalidomide, tailored to the patient's risk profile (Table 1).

## Present challenges of myeloma management in LMICs

MM is a hematological malignancy that poses significant challenges in low-resource settings due to limitations in diagnostic capabilities, treatment options and socioeconomic factors. This response provides a comprehensive analysis of the key challenges in diagnosing and managing MM in such settings, supported by evidence from relevant research papers.

### Diagnostic challenges in low-resource settings

#### Inadequate healthcare infrastructure

LMICs have either poorly developed health infrastructure or still-developing health facilities that might fall behind on essential equipment and manpower. A lack of sufficient healthcare facilities and trained personnel significantly hampers treatment delivery and patient monitoring [5].

#### Late presentation and misdiagnosis

In low-resource settings, patients with MM often present at advanced stages of the disease. This late presentation is attributed to a lack of awareness among healthcare providers and patients, as well as limited access to diagnostic facilities [18]. For instance, in Sub-Saharan Africa, skeletal-related events such as bone pain and pathological fractures often lead to misdiagnosis, as these symptoms may be mistaken for orthopedic conditions [18].

#### Limited access to diagnostic tools

The diagnosis of MM requires specialised tests such as serum protein electrophoresis (SPE), urine protein electrophoresis (UPE), bone marrow biopsy and imaging studies. However, in many low-resource settings, these diagnostic tools are either unavailable or inaccessible due to high costs or lack of trained personnel [19, 20]. For example, in Latin America, routine diagnostic studies are often not performed, leading to suboptimal risk stratification and delayed diagnosis [19].

#### Inconsistent application of standardised diagnostic criteria

In some regions, the challenge lies not in the absence of diagnostic standards, but in their consistent implementation across diverse health systems, which further complicates the timely and accurate diagnosis of MM. This is particularly evident in Sub-Saharan Africa, where the lack of data on the incidence and prevalence of MM hinders the development of region-specific diagnostic guidelines [21].

#### Knowledge gaps

Many healthcare professionals lack up-to-date knowledge on MM management, impeding the implementation of evidence-based practices [7].

### Treatment challenges in low-resource settings

#### Limited access to medications

LMICs often face challenges with medications being readily available due to manufacturing and cost issues. These countries have to rely on other countries for the medications, which might be available only at a high cost. Essential drugs, such as lenalidomide, are often unavailable, leading to reliance on alternatives like thalidomide and dexamethasone [19].

#### Limited access to novel therapies

In HICs, the treatment of MM has evolved significantly with the introduction of novel therapies such as proteasome inhibitors (e.g., bortezomib) and immunomodulatory drugs (e.g., lenalidomide). However, in low-resource settings, these drugs are often unavailable due to their high cost and limited accessibility [8, 19]. For instance, in some LICs, bortezomib-based regimens are preferred but are often limited by cost constraints, leading to the use of more affordable but less effective treatments like thalidomide and dexamethasone [8].

#### High cost of ASCT

ASCT is a standard treatment for eligible MM patients in high-resource settings. However, in low-resource settings, the high cost of ASCT and the lack of specialised facilities make it inaccessible to most patients [19, 22]. In Mexico, for example, while some patients may have access to ASCT through public healthcare, the procedure is often delayed due to financial and logistical barriers [22]. The cost of health care is particularly high in LMICs putting substantial financial burden on patient populations. The high costs of MM treatments impose substantial financial burdens on patients, adversely affecting adherence to treatment protocols [19].

#### Palliative care and supportive therapy

In many low-resource settings, the focus of MM management is often palliative care due to the advanced stage of disease at presentation. This approach includes pain management, bisphosphonates for bone lesions and supportive care for anemia and renal impairment [18]. However, even these supportive measures are often inadequate due to limited resources and lack of access to essential medications.

### Socioeconomic and healthcare system challenges

#### Economic barriers

The high cost of MM treatment, including novel therapies and ASCT, poses a significant barrier in low-resource settings. Patients often bear the financial burden of treatment out-of-pocket, leading to financial toxicity and treatment discontinuation [8, 19]. In some regions, the lack of health insurance coverage for cancer treatment further exacerbates this issue [21].

#### Healthcare infrastructure

The healthcare infrastructure in many low-resource settings is inadequate to support the diagnosis and treatment of MM. This includes a lack of specialised cancer centers, trained hematologists and diagnostic facilities [20, 23]. For example, in Sub-Saharan Africa, the scarcity of hematopathologists and modern laboratory equipment hinders the timely diagnosis and management of MM [18].

#### Comorbidities and performance status

Patients in low-resource settings often present with comorbidities such as renal impairment, anemia and infections, which can complicate treatment and worsen outcomes [24]. Additionally, poor performance status due to advanced disease at presentation further limits treatment options and reduces survival rates [24, 25].

### Strategies to address challenges in low-resource settings

#### Improving diagnostic capacity

To address diagnostic challenges, there is a need to increase access to essential diagnostic tools such as SPE, UPE and imaging studies. Training healthcare providers in the early recognition and diagnosis of MM can also improve timely referral and management [18, 21]. Establishing educational initiatives to equip healthcare workers with current knowledge on MM management can enhance the quality of care [7].

#### Enhancing access to affordable treatments

The development of resource-stratified treatment guidelines can help optimise the use of available therapies in low-resource settings. For example, the use of generic drugs and older-generation therapies such as thalidomide and dexamethasone can be effective in improving outcomes when novel therapies are unavailable [8, 19]. Introducing novel financing solutions can alleviate the economic burden on patients, thereby improving access to treatments [5]. Promoting outpatient ASCT models can enhance treatment accessibility and affordability [19].

#### Strengthening healthcare systems

Strengthening healthcare infrastructure, including the establishment of specialised cancer centers and training programs for healthcare providers, is critical to improving MM care in low-resource settings. Additionally, increasing access to palliative care and supportive therapy can improve the quality of life for patients with advanced disease [26]. Delegating specific tasks to non-specialist health workers can reduce the burden on limited specialised personnel and improve service delivery [5]. There are some examples of role of Universal Health Coverage in expanding access to treatment in MM. In India, the Ayushman Bharat–Pradhan Mantri Jan Arogya Yojana provides financial protection for economically disadvantaged populations and covers MM treatment, including bortezomib-based regimens and ASCT at designated tertiary centers [27]. In Thailand, the Universal Coverage Scheme includes coverage for key cancer therapies, though access to ASCT may still be centralised and capacity-limited [28]. In Mexico, while public insurance schemes like Seguro Popular previously covered basic cancer treatment, MM-specific drug access and ASCT coverage have remained inconsistent and often delayed due to reimbursement and logistical constraints [9]. In South Africa, public coverage for cancer care is limited and high-cost therapies like ASCT and novel agents remain largely inaccessible in the public sector [29] (Table 2).

#### Advocacy and policy interventions

Advocacy efforts are needed to raise awareness about MM and the challenges faced in low-resource settings. Policy interventions, such as increasing funding for cancer care and expanding health insurance coverage, can help reduce the financial burden on patients and improve access to essential treatments [21]. While these strategies hold promise for addressing treatment challenges in LMICs, broader systemic barriers – such as political instability and socioeconomic disparities – remain significant impediments. Tackling these structural issues will be essential for achieving sustainable and equitable improvements in MM care.

In response to the growing recognition of global disparities in MM care, several international bodies have developed resource-stratified treatment guidelines to aid clinicians practicing in LMICs. The NCCN Harmonised Guidelines™, for example, provide tiered treatment algorithms tailored for Sub-Saharan Africa and South Asia, recommending regimens such as VTD or CTD in basic settings, while incorporating VRd in regions where generic lenalidomide is accessible [30]. Similarly, the IMWG has proposed flexible, resource-adjusted treatment pathways for diagnosis, risk stratification and therapy selection, emphasising cost-effectiveness without compromising core principles of disease management [26]. These are complemented by broader frameworks from ASCO that focus on infrastructure, supportive care and system-level planning in LMICs. Incorporating and adapting these stratified guidelines into national protocols can help bridge gaps in care and promote standardised, evidence-based treatment delivery across diverse economic settings [31].

## Consideration of emerging immunotherapies in resource-limited settings

Recent advances in MM treatment have introduced powerful immunotherapies, notably chimeric antigen receptor (CAR) T-cell therapy and bispecific T-cell engagers (BiTEs), which have significantly improved outcomes in relapsed or refractory disease. CAR T-cell therapies, such as idecabtagene vicleucel and ciltacabtagene autoleucel, have demonstrated high response rates and durable remissions in heavily pretreated patients [32, 33]. Similarly, bispecific antibodies targeting B-cell maturation antigen, including teclistamab and elranatamab, have shown promise in early-phase clinical trials, offering an off-the-shelf immunotherapy alternative to CAR T cells [34,35].

However, the translation of these therapies to LMICs remains a formidable challenge. Both CAR T-cell therapies and BiTEs require specialised infrastructure, including cell manufacturing facilities, intensive supportive care capabilities and trained personnel to manage immune-related toxicities such as cytokine release syndrome and neurotoxicity [36]. Furthermore, the high financial cost associated with these treatments – often exceeding six figures in USD – renders them inaccessible to most healthcare systems in LMICs without significant external support or policy intervention [36].

While the integration of these therapies into resource-constrained settings may not be immediately feasible, their growing role in MM management warrants acknowledgment. As global clinical trial networks expand and biosimilar innovation progresses, future strategies should prioritise equitable access to these transformative agents. In the interim, LMICs may consider participation in international collaborative trials or technology transfer initiatives aimed at expanding treatment access and training [36].

### Drug price dynamics in LMICs

In recent years, the cost landscape for MM therapies has undergone notable changes in several LMICs, primarily driven by the availability of generic formulations and negotiated procurement agreements. Lenalidomide, previously a cost-prohibitive immunomodulatory agent, is now accessible in generic form in countries such as India, Bangladesh and parts of Latin America at a fraction of its original cost, which is enabling broader use of standard regimens like VRd in frontline settings [8]. Similarly, generic pomalidomide is emerging in select markets, though its uptake remains limited due to ongoing regulatory and supply chain barriers. Carfilzomib, while still costly in most LMICs, is increasingly available through public-private access programs and named-patient schemes in middle-income countries [25]. These price shifts are gradually reshaping treatment algorithms, allowing more patients to receive guideline-recommended therapies. However, significant disparities persist across regions depending on national healthcare financing models, patent laws and local manufacturing capabilities. Sustainable access to affordable MM drugs will require coordinated policy interventions, support for regional generic manufacturing and expanded participation in global pooled procurement mechanisms [5].

## Discussion

The increasing global burden of MM presents a significant challenge for healthcare systems, particularly in LMICs. One of the central challenges is the disparity in diagnostic capabilities. In HICs, early and accurate diagnosis is facilitated by advanced diagnostic tools and standardised criteria, which enable timely intervention. In contrast, LMICs often face delays due to limited access to sophisticated diagnostic tests such as SPE, UPE and imaging studies. These limitations not only contribute to late presentation but also hinder effective risk stratification, ultimately affecting treatment outcomes.

Therapeutically, the divergence in available treatment options between high-income and low-income settings is stark. While HICs routinely implement intensive induction regimens, ASCT and maintenance therapies with novel agents, LMICs are frequently restricted to more affordable regimens such as bortezomib- or thalidomide-based combinations. Although these alternatives offer a pragmatic solution given budgetary constraints, they often do not match the efficacy of newer, more expensive therapies. Thus, the challenge lies in balancing cost-effectiveness with optimal clinical outcomes.

In high-income settings, the median overall survival (OS) for newly diagnosed MM patients receiving triplet or quadruplet induction followed by ASCT and maintenance therapy can now exceed 7–10 years for standard-risk patients [14]. In contrast, studies from LMICs report significantly lower survival figures. For example, real-world data from a tertiary center in Mexico demonstrated a median OS of 2.5 years, primarily due to delayed diagnosis, limited access to ASCT and restricted use of novel agents [22]. A 2022 survey of MM treatment centers in Latin America reported 5-year survival rates below 40%, compared to over 60% in Western Europe and North America [20]. A study from India showed improved outcomes with VRd-based induction, reporting a 3-year progression-free survival of 55% in transplant-eligible patients, yet this still lags behind HIC benchmarks due to lower transplantation rates and financial toxicity [8].

The financial burden of MM treatment in LMICs cannot be overstated. The high cost of novel therapeutic agents and procedures such as ASCT creates an insurmountable barrier for many patients, leading to significant economic toxicity. In many cases, patients must shoulder out-of-pocket expenses, which can exacerbate disparities in treatment adherence and OS. Moreover, inadequate health insurance coverage further compounds these issues, leaving vulnerable populations with limited recourse. In addition to economic constraints, systemic healthcare infrastructure limitations, including the scarcity of specialised cancer centers and trained personnel, create additional hurdles. The lack of dedicated hematopathologists and modern laboratory facilities often results in misdiagnosis or underdiagnosis, delaying critical interventions and ultimately diminishing the prospects for long-term remission.

Given these challenges, the review advocates for a multi-pronged approach aimed at improving MM care in resource-limited settings. Enhancing diagnostic capacity through training programs and improved access to essential tests is paramount. Equally important is the development of resource-stratified treatment guidelines that prioritise the use of cost-effective therapies without compromising the quality of care. In this context, leveraging generic medications and adapting outpatient models for procedures like ASCT may offer feasible solutions.

Strengthening healthcare systems through targeted investments in infrastructure and human resources is critical. Establishing specialised centers and fostering partnerships between the public and private sectors could mitigate many of the current limitations. Additionally, policy interventions, including increased funding for cancer care and expanded health insurance coverage, are necessary to reduce the financial strain on patients and improve treatment accessibility.

### Future directions

While the strategies outlined provide a framework for addressing current challenges, ongoing research and innovation remain essential. Future studies should focus on the efficacy of adapted treatment protocols in LMICs, evaluating long-term outcomes and quality-of-life measures. Furthermore, there is a need for robust clinical trials conducted in diverse resource settings to validate the effectiveness of cost-reduced interventions. Such evidence would be instrumental in shaping global guidelines that are both effective and adaptable to varying economic realities.

In summary, optimising MM therapy in resource-limited settings requires a comprehensive approach that addresses diagnostic, therapeutic and systemic challenges. Through collaborative efforts among clinicians, policymakers and researchers, it is possible to develop sustainable models of care that improve outcomes and reduce global health inequities in the management of MM.

## Conclusion

The diagnosis and management of MM in low-resource settings face multiple challenges, including late presentation, limited access to diagnostic tools, novel therapies and socioeconomic barriers. Addressing these challenges requires a multifaceted approach that includes improving diagnostic capacity, enhancing access to affordable treatments, strengthening healthcare systems and advocating for policy changes to support care of MM patients in resource-constrained settings. Future efforts should prioritise collaborative research and the development of innovative models of care that are sensitive to the unique constraints of low-resource environments. Ultimately, addressing these multifaceted issues is imperative for improving patient outcomes, reducing global health inequities and establishing sustainable frameworks for the optimal management of MM worldwide.

## Conflicts of interest

The authors declare no conflicts of interest.

## Funding

The authors declare that no specific grant or funding from any public, commercial or not-for-profit agency was received for the preparation of this article.

## Figures and Tables

**Table 1.** Comparative summary of MM treatment strategies in LICs and HICs.

Treatment component	LICs	HICs
First-line therapy	Bortezomib- or thalidomide-based regimens such as VCD (bortezomib, cyclophosphamide, dexamethasone), VTD or CTD (cyclophosphamide, thalidomide, dexamethasone). Selected for affordability.	Triplet regimens such as VRd. In high-risk cases, quadruplet regimens (e.g., Dara-VRd) are used per international guidelines.
Maintenance therapy	Thalidomide is commonly used due to its low cost.	Lenalidomide for standard-risk; bortezomib + lenalidomide for high-risk patients.
ASCT	Rarely performed due to high cost and lack of infrastructure.	Standard of care for eligible patients post-induction. Conducted routinely in specialised centers.
Treatment for relapsed/refractory MM	Cost-effective options like CTD preferred. Bortezomib used if affordable.	Wide access to novel agents (e.g., carfilzomib, daratumumab) guided by molecular risk and treatment history.
Supportive care	Basic measures like bisphosphonates, antibiotics, transfusions; limited by supply and personnel.	Integrated supportive care including bone protection, infection control and symptom management per ASCO/ESMO recommendations.
Healthcare delivery strategy	Emphasises use of generics, task-shifting to non-specialist providers and improving infrastructure through cost-sensitive models.	Focuses on precision medicine, guideline-based therapy and continuous care in multidisciplinary settings.

This table outlines key differences in treatment approaches for MM between LICs and HICs, highlighting disparities in therapeutic regimens, access to transplantation, supportive care and strategic healthcare focus.

**Table 2.** Key challenges and strategies for MM treatment in low-resource settings.

Challenge	Description	Strategies
Late presentation	Patients often present with advanced disease due to lack of awareness and diagnostics.	Improve awareness and access to diagnostic tools; train healthcare providers.
Limited access to novel therapies	High cost and unavailability of novel therapies like bortezomib and lenalidomide.	Use of generic drugs and older therapies; advocate for affordable treatment options.
High cost of ASCT	Limited access to ASCT due to high cost and lack of specialised facilities.	Increase funding for ASCT; develop public-private partnerships for cancer care.
Economic barriers	Financial toxicity and lack of health insurance coverage.	Expand health insurance; reduce out-of-pocket costs for patients.
Healthcare infrastructure	Inadequate infrastructure and lack of trained personnel.	Strengthen healthcare infrastructure; train hematologists and support staff.
